# Effects of Micronutrients on the Growth and Phytochemical Composition of Basil (*Ocimum basilicum* L.) in the Field and Greenhouse (Hydroponics and Soil Culture)

**DOI:** 10.3390/plants13172498

**Published:** 2024-09-06

**Authors:** Hamid Aghamirzaei, Hasan Mumivand, Abdollah Ehtesham Nia, Mohamad Reza Raji, Alfred Maroyi, Filippo Maggi

**Affiliations:** 1 Department of Horticultural Sciences, Faculty of Agriculture, Lorestan University, Khorramabad P.O. Box 465, Iran; 2 Department of Botany, University of Fort Hare, Alice 5700, South Africa; 3 Chemistry Interdisciplinary Project (ChIP) Research Center, School of Pharmacy, University of Camerino, 62032 Camerino, Italy

**Keywords:** essential oil, phenol, flavonoid, antioxidant activity

## Abstract

The current research was conducted to compare the growth, yield, and phytochemical composition of basil (*Ocimum basilicum*) in the open field and the soil and hydroponic cultivation in a greenhouse. Furthermore, the effect of foliar spraying of micronutrients on this crop was also evaluated. In each of the cultivation systems, foliar spraying of one micronutrient, either iron sulfate (Fe), zinc sulfate (Zn), copper sulfate (Cu), manganese sulfate (Mn), or boric acid (B), at a concentration of 0.1% was applied in a randomized complete block design. Plants grown in the hydroponic system had higher yield and biomass. The concentration of the elements K, Ca, Mg, N, P, Mn, Fe, B, and Zn in the leaves of hydroponic plants was higher. Contrarily, plants cultivated in the field showed higher stem dry weight, essential oil content, phenolic and flavonoid content, and antioxidant activity. The level of methyl chavicol was higher in the hydroponic culture, but the level of 1,8-cineole was much lower in this cropping system. Foliar spraying of Cu, Mn, Zn, Fe, or B significantly increased leaf dry weight and anthocyanin content. In field conditions, the highest levels of phenolics, flavonoids, and antioxidant capacity were observed with Zn or Mn application. In the hydroponic system, foliar spraying of Zn or B led to the highest antioxidant capacity, and total phenolic and flavonoid contents. Overall, the basil plants cultivated in the field showed higher bioactive ingredients. However, the essential oil of plants cultivated in the hydroponic system had a higher economic value due to its higher percentage of methyl chavicol.

## 1. Introduction

Basil (*Ocimum basilicum* L.) is an important memeber of the Lamiaceae family, widely used as a medicinal plant, spice, and fresh vegetable. The bioactive ingredients of this plant are used to treat flatulence and anorexia and aid digestion [1]. Moreover, its extract and essential oil are widely used in the food industry as a flavoring agent [2]. Phenolics in vegetables and plants are the main bioactive compounds known for their antioxidant properties [3]. Basil essential oil contains monoterpenoids (limonene, camphor, linalool, geraniol) and phenylpropanoids (eugenol, methyl chavicol), which are also responsible for the medicinal properties of this plant [4]. Essential oils are complex mixtures of volatile compounds, whose quantity and quality are affected by various factors such as genetics, climatic and geographical conditions (elevation of habitat, quantity and quality of light, temperatures, availability of water, salinity, soil characteristics), and agricultural operations (access to macroelements and microelements, irrigation regimes, tillage) [5,6,7].

There are different types of cultivation systems, which differ from each other in terms of ideology or technology. In recent years, due to the shortages of water or the salinity of water and soil in many regions of the world, the focus has been directed to the production of high-quality horticultural crops under controlled conditions, and new alternative techniques have been developed to produce these plants at a high density [8]. Outdoor agriculture faces many challenges, including soil-borne diseases, lack or inadequate levels of organic matter in the soil, infeasibility of cultivating certain products, lack of precise control of nutrients at different stages of plant growth in the soil, difficulty of weed control, low efficiency of water consumption, and impossibility of controlling environmental conditions. Therefore, despite the availability of soil resources and land suitable for agriculture in some areas, due to the above challenges, it is necessary to use alternative methods to produce crops. A suitable method to achieve high agricultural production in small areas is to use controlled environment agriculture so that while increasing the production per unit area, high-quality crops in the desired quantities can be produced throughout the year [9,10]. The increasing interest in soilless cultivation is due to the many problems associated with the open field cultivation, such as soil diseases, weeds, and issues related to rotation [11,12]. In addition, the lack of water and the impossibility of precisely controlling plant nutrition in soil systems have led to an increase in the production of crops through different methods of soilless cultivation in recent decades [13,14]. Some of the advantages of hydroponic cultivation are the lack of contamination with soil diseases, higher crop yields, no dependence on soil quality, and the ability to precisely control water and nutrients [9,13].

Comparing the yield and quality of crops in different cropping systems is often challenging due to the intricate interplay between genotype, plant physiology, and the environment. Nevertheless, it is crucial to carry out controlled comparisons in order to pinpoint the most efficient and effective food production systems [10]. Maliqa et al. [12] stated that in the hydroponic cultivation system, the rate of photosynthesis and plant yield were significantly higher than in the soil-based system. However, a significant decrease in dry matter was observed in hydroponic conditions. A comparison of hydroponic and field cultivation of *Carlina acaulis* L. showed that the antioxidant capacity and content of carlina oxide were significantly higher in field plants. However, the flavonoid content in the leaves of plants grown in both systems was similar [15]. In the comparison of tissue culture plants, field culture, and hydroponic culture of *Acmella oleracea* (L.) R.K.Jansen, the content of total phenolics, total flavonoids, and antioxidants in plants from hydroponic culture was higher than in field-grown plants and in callus from tissue culture [14].

The concentration of macroelements and microelements in the soil is one of the key factors in the production and accumulation of bioactive substances in medicinal plants, and the level of their absorption has a great effect on the biosynthesis of medicinal compounds [16,17]. Among the micronutrients, Zn plays a vital role in cellular functions such as indole acetic acid (IAA) synthesis, protein metabolism, and photosynthesis, and its deficiency causes thickening of leaves, premature loss of foliage, and stunted growth. Fe plays a vital role in chlorophyll synthesis, carbohydrate production, and cellular respiration. Fe deficiency results in interveinal chlorosis of younger leaves, commonly known as iron chlorosis [18]. B plays a crucial role in stabilizing certain components of the cell wall and plasma membrane; it contributes to the strengthening of cell division and supports the metabolism of nucleic acids, carbohydrates, proteins, auxins, and phenolics. Generally, plants are prone to Zn, Fe, and B deficiency in alkaline soil with a coarse texture and low organic matter. The deficiency of Zn, Fe, and B (or their insufficient fertilization) may be a significant reason for low productivity [6,19]. In calcareous soils, Cu, Fe, Mn, and Zn are less available because they precipitate in the soil to form carbonates or bicarbonates, and they are also less available in soils with high organic matter [20]. Micronutrients such as Fe, Cu, Mn, and Zn are key factors for the activity of various enzymes in plants, especially those affecting the biosynthesis of secondary metabolites [17].

Inadequate availability of organic matter in the soil, high salinity, low humidity, high bicarbonate ions in irrigation water, high lime and soil pH, and imbalanced use of fertilizers lead to deficiencies of micronutrients in plants. Deficiency of nutrients is common in almost all farms in the world and its level varies in different regions and from plant to plant [21]. Foliar spraying of nutrients is a common method of meeting the nutritional demands of higher plants, which is more efficient than soil application in poor soils in terms of nutrient availability [6,22]. Spraying basil with Zn chelates under salinity stress conditions improved the growth and yield of the plant, increased the percentage and yield of essential oil, and raised the levels of linalool and methyl chavicol, the dominant components of basil’s essential oil [23]. The application of Zn (50 µM) increased the concentration of polyphenols, carotenoids, and antioxidant capacity of the two basil varieties by 14.57, 19.76, and 33.72%, respectively, compared to the control [24]. B or Zn foliar application improved the content of total phenolics, essential oil content and yield, and antioxidant capacity of *Satureja khuzistanica* Jamzad. Furthermore, the application of B increased the production of carvacrol in the essential oil [6].

In addition to having an essential role in the growth and yield of medicinal plants and vegetables, micronutrients are effective in increasing their quality and nutritional value. Considering the importance of basil as a fresh vegetable and its special use in the nutraceutical industry, there is a high demand for consuming this vegetable throughout the year. Therefore, to address the requirements of the growing human population, there is a need to develop new agricultural systems. In addition, growth rates, yields, and crop quality vary across modern cultivation systems. A review of the literature also shows that no comparison has been made so far between the modern systems of greenhouse cultivation and field cultivation of basil. The primary objective of the present study was to compare the growth, yield, phytochemical characteristics, and nutritional elements of basil cultivated in different environments, specifically in the open field and the soil and hydroponic cultivation in greenhouses. Furthermore, the study aimed to assess the impact of foliar spraying of micronutrient elements on the growth and development of basil plants.

## 2. Results

### 2.1. Yield and Biomass

The analysis of variance (Table S1) showed that the effect of the cultivation system on all parameters related to yield and biomass, including plant height, leaf area, stem dry weight, and leaf dry weight, was significant (*p* ≤ 0.01). All traits related to yield and biomass were significantly affected by the foliar spraying of micronutrients. The effect of cultivation system × foliar spraying was also significant on plant height, stem dry weight, and leaf area.

The mean comparison of the effect of the cultivation system × foliar spraying of micronutrients (Table 1) demonstrated that the highest plant height was found in the hydroponic system with the B, Cu, Mn, and Zn foliar spray treatments (60.93 ± 3.56, 60.45 ± 5.32, 58.81 ± 3.24, and 58.05 ± 5.33 cm, respectively). Contrarily, the lowest plant height was observed in the control treatment and in the field (31.07 ± 1.40 cm). Overall, plants grown in the hydroponic system were taller than in other systems, and the lowest plant height was obtained in the field cultivation. In field cultivation, the highest plant height was obtained with Fe foliar spray treatment, while in soil and hydroponic greenhouse cultivations, plants treated with B showed the highest plant height. The highest leaf area was obtained in hydroponic cultivation with Mn spraying (15.33 ± 2.42 cm^2^), which was not significantly different from those in B, Cu, Zn, and Fe treatments in the hydroponic system. The lowest leaf area was observed in the field cultivation with the control treatment (5.87 ± 0.62 cm^2^). In general, greenhouse-grown plants, especially in the hydroponic system, had a higher leaf area. The highest stem dry weight (14.56 ± 2.22 g) was found in the field cultivation with Fe foliar application, which did not have a significant difference from those of Cu, Zn, B, or Mn treatment in the field cultivation and Cu, Fe, or Zn treatment in the soil greenhouse cultivation. The lowest stem dry weight was obtained in the soil greenhouse cultivation system with no foliar spray (4.25 ± 0.54 g), which was not significantly different from the no foliar spray treatment in the hydroponic cultivation system. The plants grown in the greenhouse showed lower stem dry weight, especially compared to the field cultivation (Table 1).

**Figure 1:**
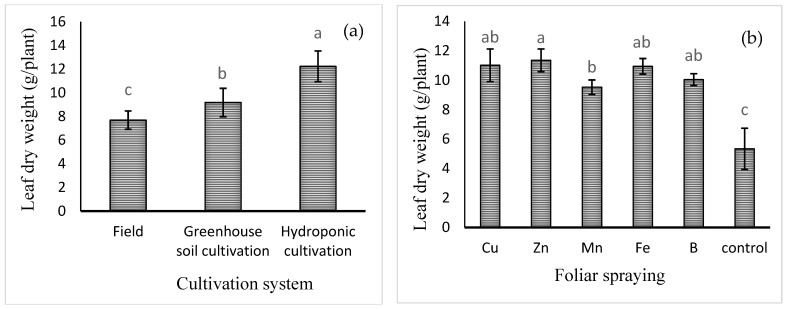
Mean comparison of the effects of (**a**) cultivation system and (**b**) foliar spraying of micronutrients on basil leaf dry weight. Different lowercase letters indicate significant differences among different treatments.

Mean comparison of the effect of cultivation system on leaf dry weight (Figure 1a) showed that the highest leaf dry weight was observed in hydroponic cultivation (12.23 ± 1.33 g) and the lowest in field cultivation (7.69 ± 0.78 g). Furthermore, the mean comparisons (Figure 1b) also showed that the highest leaf dry weight was recorded for Zn foliar spray (11.35 ± 0.77 g), which was 106% higher compared to the control treatment (no foliar spray), although not significantly different from that of Cu, Fe, or B treatment. The lowest leaf dry weight was obtained in the control treatment (5.34 ± 1.42 g).

### 2.2. Macronutrients

The analysis of variance of the effects of cultivation system and foliar spray of micronutrients on the macronutrients of basil is shown in Table S2. The effect of the cultivation system on the concentration of all macronutrients, including N, P, Ca, K, and Mg, was significant. Contrary to this, the simple effect of foliar application of micronutrients and the interactive effect of cultivation system and foliar application of micronutrients were not significant on all of the macronutrients. The mean comparisons of the effect of the cultivation system on the levels of macronutrients in basil leaves are shown in Table 2. The highest levels of all macronutrients, including N (4.06 ± 0.67%), K (1.25 ± 0.28%), P (0.6 ± 0.17%), Mg (0.29 ± 0.03%), and Ca (0.58 ± 0.06%), were found in the hydroponic cultivation system, while the lowest were found in the field cultivation system.

### 2.3. Micronutrients

The analysis of variance (Table S2) revealed that the effects of the cultivation system and foliar spraying of micronutrients were significant on the levels of micronutrients (Cu, Fe, Zn, Mn, and B). Contrarily, the interaction effect of the cultivation system and micronutrient spray on the concentration of any of the micronutrients was not significant. The highest levels of Zn, Fe, Mn, and B (53.36 ± 3.94, 74.08 ± 8.75, 94.3 ± 8.63, and 5.66 ± 1.44 µg/g, respectively) were found in the hydroponic system. The lowest levels of all these elements were observed in field cultivation. However, the highest level of Cu (7.94 ± 1.48 µg/g) was observed in the soil culture of the greenhouse and the lowest in the hydroponic system and the field (Table 2).

The mean comparison of the influence of foliar spraying of micronutrients on the levels of micronutrients in the aerial parts of basil is shown in Table 3. The highest level of Cu (12.91 ± 2.38 µg/g) was observed in the treatment of Cu foliar spray, which was 83.64% higher compared to the control (water spray). The lowest level of Cu (6.35 ± 1.46 µg/g) was observed following Fe foliar spray. The highest level of Zn (54.02 ± 4.75 µg/g) was observed in the zinc foliar spray treatment, which was 41.71% higher compared to the control treatment (water spray). The lowest level of Zn was obtained in the Cu foliar spray treatment. Fe foliar application increased the level of Fe in the aerial parts of basil by 56.54% compared to the control. The highest level of Fe (94.95 ± 8.37 µg/g) was observed in the treatment under Fe application. The foliar spray of Mn significantly increased the content of Mn in the aerial parts of basil. The highest level of Mn (90.30 ± 8.63 μg/g) was obtained in the treatment with Mn foliar spray, which showed an increase of 74.86% compared to the treatment of water spray. The highest level of B (7.05 ± 2.08 μg/g) was observed in the treatment of B spray, which was 44.05% higher compared to the control treatment (Table 3).

### 2.4. Phytochemistry

The analysis of variance (Table S1) revealed that the simple effects of the cultivation system and foliar spraying of micronutrients on the levels of total anthocyanins, phenolics, and flavonoids, antioxidant properties by FRAP and DPPH methods, and essential oil content and yield were significant. The effect of cultivation system × foliar spraying was also significant on total phenolics and flavonoids, antioxidant properties by FRAP and DPPH methods, and essential oil yield.

#### 2.4.1. Anthocyanins

**Figure 2:**
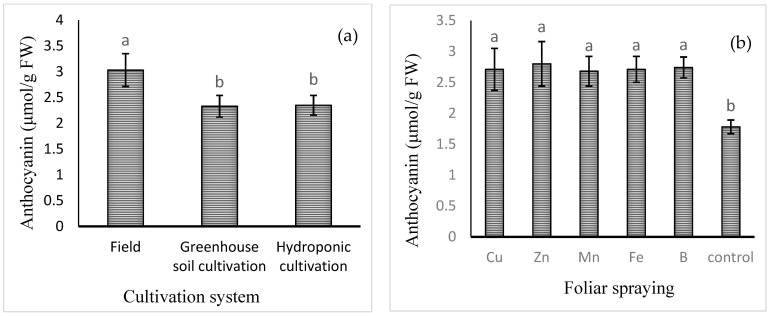
Mean comparison of the effects of (**a**) cultivation system and (**b**) foliar spraying of micronutrients on anthocyanin content of basil. Different lowercase letters indicate significant differences among different treatments.

The mean comparisons of the effect of the cultivation system on the levels of anthocyanins are shown in Figure 2a. The highest content of anthocyanin (3.03 ± 0.32 μmol/g FW) was found in field cultivation and the lowest in the hydroponic (2.33 ± 0.21 μmol/g FW) and soil greenhouse (2.35 ± 0.19 μmol/g FW) cultivation systems. Among the foliar applications of different micronutrients, the highest level of anthocyanin was found in the Zn treatment (2.80 ± 0.36 μmol/g FW), which was not significantly different from that of the B, Cu, Fe, or Mn treatment. The lowest level of anthocyanin was observed in the control treatment (1.78 ± 0.11 μmol/g FW).

#### 2.4.2. Total Phenolic and Flavonoid Content

The mean comparisons of the effect of the cultivation system × foliar spray on the content of total phenolics showed that the highest level (16.54 ± 2.42 mg GAE/g DW) was observed in field cultivation under the Zn foliar application. The lowest level of total phenolics was found under the hydroponic cultivation system with the control, Cu, and Mn treatments (1.57 ± 0.42, 2.4 ± 0.62, and 2.64 ± 0.18 mg GAE/g DW, respectively). The highest level of flavonoids (9.65 ± 2.01 mg QU/g DW) was observed in field cultivation with Zn foliar treatment. The lowest level of flavonoids (0.64 ± 0.12 mg of QU/g DW) was observed in the hydroponic cultivation system with no foliar spray, which was not significantly different from the Cu or Mn foliar spray in the hydroponic cultivation system. Generally, the plants grown in the field possessed higher contents of total phenolics and flavonoids compared to greenhouse cultivation (Table 1).

#### 2.4.3. Antioxidant Activity

The mean comparisons of the cultivation system × foliar spraying of micronutrients showed that the highest antioxidant activity, measured with the DPPH method, was found in the field cultivation system with foliar spray of Zn, Mn, or B (with IC50 equal to 5.41 ± 1.1, 5.84 ± 0.87, and 5.99 ± 1.42 mg/mL). The lowest antioxidant activity was recorded for hydroponic culture with water spray or Cu foliar spray (with IC_50_ equal to 10.48 ± 2.01 and 10.25 ± 1.7 mg/mL, respectively). IC_50_ represents the concentration of the extract that inhibits free radicals by 50%, so a lower IC_50_ indicates a higher antioxidant property. The highest level of antioxidants, measured with the FRAP method, was observed in the field cultivation system under the foliar spray of Zn, Mn, or B (7.26 ± 1.02, 7.15 ± 1.14, or 7.08 ± 1.13 mmol Fe/g DW, respectively). In addition, it was found that plants grown in the field showed higher antioxidant activity, regardless of the measurement method (Table 1).

#### 2.4.4. Essential Oil Content and Yield

**Figure 3:**
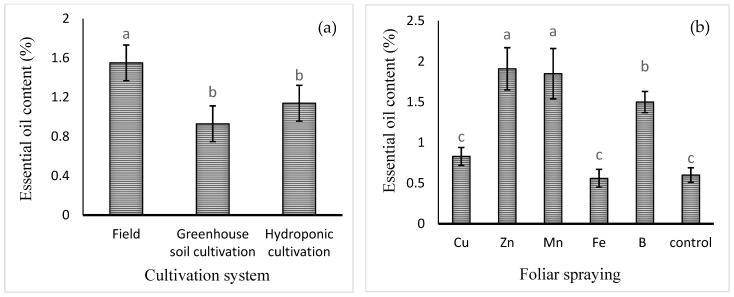
Mean comparison of the effects of (**a**) cultivation system and (**b**) foliar spraying of micronutrients on basil essential oil content (%). Different lowercase letters indicate significant differences among different treatments.

The mean comparisons of the effect of the cultivation system on the content of basil essential oil (expressed in eprcentage) are shown in Figure 3a. The highest level of essential oil was obtained in field cultivation (1.55 ± 0.23% *w*/*w*). Foliar application of micronutrients, except for that of Fe or Cu, increased the essential oil percentage. Foliar application of Zn, Mn, or B increased the content of essential oil by 218, 208, and 150%, respectively, compared to the control (Figure 3b). The mean comparisons of the effect of the cultivation system × foliar spray on essential oil yield are shown in Table 2. The highest essential oil yield (0.3 ± 0.05 g/plant) was noted in field cultivation with Zn foliar spray, though this was not significantly different from the yield in hydroponic culture treated with Mn foliar spray. In the comparison between cultivation systems, it was found that there was no significant difference between field and hydroponic cultivation in terms of essential oil yield.

#### 2.4.5. Essential Oil Compounds

GC-MS analysis of basil essential oil identified 31 compounds (Table 4). Methyl chavicol (average: 43.08%), linalool (average: 25.46%), and *epi*-α-cadinol (average: 9.2%) were identified as the main components of basil essential oil. In addition, germacrene-D, α-bergamotene, 1,8-cineole, and γ-cadinene were other important components of the essential oil. The effect of the cultivation system on essential oil compounds, including 1,8-cineole, linalool, methyl chavicol, α-bergamotene, γ-cadinene, δ-cadinene, spathulenol, 1,10-di-epi-cubenol, and epi-α-cadinol, was significant. The percentages of 1,8-cineole, α-bergamotene, γ-cadinene, caryophyllene oxide, 1,10-di-*epi*-cubenol, and *epi*-α-cadinol changed significantly following the foliar application of micronutrients. The effect of the cultivation system × micronutrient foliar application was also significant in the case of 1,8-cineole, methyl chavicol, α-bergamotene, γ-cadinene, caryophyllene oxide, 1,10-di-*epi*-cubenol, and *epi*-α-cadinol (Table S1).

**Figure 4:**
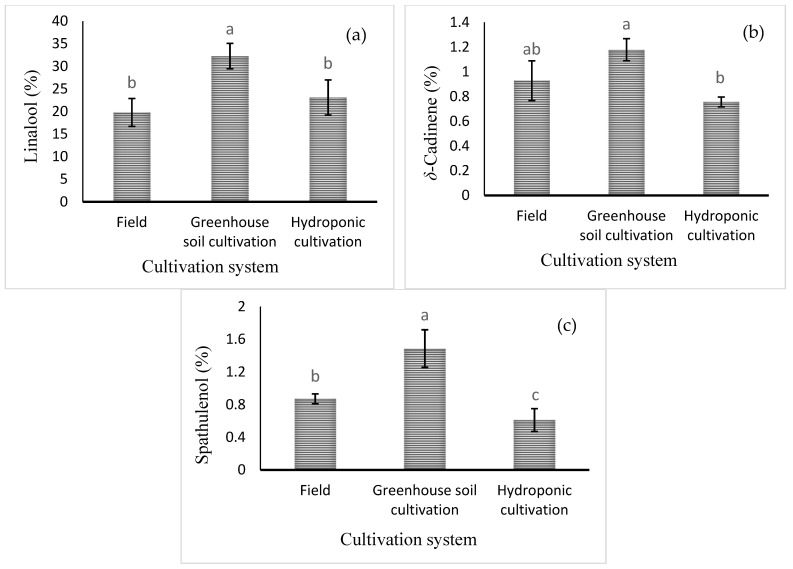
Mean comparison of the effect of cultivation system on (**a**) linalool, (**b**) δ-cadinene, and (**c**) spathulenol relative percentages in basil essential oil. Different lowercase letters indicate significant differences among different treatments.

Mean comparisons of the effect of the cultivation system are shown in Figure 4. The highest levels of linalool, δ-cadinene, and spathulenol (32.27 ± 2.8%, 1.18 ± 0.09%, and 1.48 ± 0.23%, respectively) were obtained in the greenhouse soil culture (Figure 4). Mean comparisons of the effect of the cultivation system × foliar spraying are shown in Table 5. The highest percentage of 1,8-cineole was noted in the field cultivation under Cu and Zn treatments (6.29 ± 1.08 and 6.8 ± 1.11%, respectively). However, the level of 1,8-cineole in hydroponic culture was very low, regardless of the foliar spraying treatment. The highest level of methyl chavicol, the main component of basil essential oil, was observed in all treatments with foliar application of micronutrients in hydroponic cultivation. However, there was no significant difference with Mn or B foliar spraying in the hydroponic cultivation. The level of methyl chavicol in hydroponic culture was significantly higher than in soil culture in the greenhouse and in the field. The highest percentage of caryophyllene oxide (2.26 ± 0.14%) was observed under Fe foliar application in field cultivation. Furthermore, the highest percentage of α-bergamotene (3.76 ± 1.01%) was observed in hydroponic culture with water spraying. The highest level of δ-cadinene was observed in the control (2.65 ± 0.38%) and/or B spray (2.2 ± 0.23%) treatments in the greenhouse soil culture and in the Fe foliar treatment in the field culture (2.03 ± 0.28%). However, the highest percentage of 1,10-di-*epi*-cubenol (5.1 ± 1.01%) was obtained in the field cultivation system with Fe application. The highest percentage of *epi*-α-cadinol (24.01 ± 3.78%) was obtained in greenhouse soil culture with Zn foliar application. Finally, the percentage of *epi*-α-cadinol in greenhouse soil culture was much higher than in field soil culture and hydroponic culture (Table 5).

## 3. Discussion

In the present study, basil plants cultivated in a hydroponic system had higher plant height, leaf dry weight, and leaf area. Contrarily, basil plants grown in the field were woodier than those grown in the soil in the greenhouse and in the hydroponic system, resulting in a higher stem dry weight. The better growth and higher yield of basil in the hydroponic system may be attributed to better aeration of the root zone, which leads to better plant root activity. In addition, the presence of sufficient nutrients in the growing media and increased absorption by the roots leads to the improvement of the photosynthetic efficiency, and, therefore, increases the growth and biomass of the plant. The higher relative humidity of the greenhouse and optimal absorption of water by the roots in the hydroponic system also improve the water balance of the plant and, therefore, increase the division and enlargement of the leaf mesophyll cells (higher leaf area) and increase the content of chlorophyll [25]. The results of the present study also showed that the uptake of nutrients such as K, Ca, Mg, N, P, Mn, Fe, B, and Zn in the hydroponic cultivation system was much better than in the greenhouse soil cultivation and field cultivation systems. Therefore, the better nutritional status of the plant in hydroponic conditions leads to higher growth and yield. The increase in the absorption of mineral elements in soilless cultivation can be due to the optimal concentration of these elements, increasing root access to nutrients due to the suitable pH of the nutrient solution and culture medium. In the case of less mobile elements such as Ca, the absorption is strongly influenced by the humidity of the substrate and the access of the plant roots to water. The optimal availability and solubility of Ca in the culture medium may result from the improvement of the water content of the medium, thereby increasing Ca absorption in the soilless system [26,27]. In a study comparing the vegetative and physiological characteristics of *Aloysia citriodora* Palau in two cultivation systems (soil and hydroponics), it was found that the plants grown in the hydroponic system had a higher stem dry weight, root dry weight, plant dry weight, shoot length, and shoot fresh weight compared to soil grown plants [28]. In another trial, lavender, garden thyme, and marigold plants were compared in hydroponic/air and soil cultivation systems in the greenhouse. The results showed that plant growth and yield indices were higher in hydroponic and air cultivation systems than in a soil cultivation system [29]. 

Different cultivation systems affect the concentration of bioactive compounds in medicinal plants differently [30]. In the present study, the content of total phenolics, flavonoids, and anthocyanins, the antioxidant capacity of the extract, and the percentage of basil essential oil in the field were higher than in greenhouse cultivation systems (soil and hydroponics). However, there was no significant difference in the yield of essential oil between field cultivation and hydroponic systems. Considering that essential oil yield depends on both the percentage of essential oil and plant biomass [7], the high yield of essential oil in the field and the high yield of plant biomass in the hydroponic system led to similar essential oil yield. Therefore, there was no significant difference between the two systems. Contrarily, Giurgiu et al. [29] reported that lavender and garden thyme plants grown in hydroponic systems had 20–30% more essential oil yield than those grown in the field. These differences in the responses of plants could be due to factors such as the inherent characteristics of the species, plant origin, experimental conditions, and different types of hydroponic systems [5,30].

Phenolic compounds act effectively as hydrogen donors and are considered important antioxidants [31]. Given the higher levels of phenolics, flavonoids, and anthocyanins in basil cultivated in the field, the high antioxidant activity in field-grown plants is unsurprising [32]. Studies have shown that lack of water combined with high light intensity (field conditions) leads to the closure of the stomata, as a result of which the absorption of carbon dioxide and its stabilization is reduced. Following this, the consumption of NADPH+H^+^ to stabilize carbon dioxide decreases during the Calvin cycle, which leads to the accumulation of high levels of NADPH+H^+^ in the cell. In such conditions, the production of highly reduced natural compounds (secondary metabolites) that require high consumption of NADPH+H^+^ increases [33]. In fact, the biosynthesis of secondary metabolites may be an auxiliary system to release excess energy in conditions of stomatal closure in plants under stress. Therefore, regardless of their ecological effects, secondary metabolites can play a vital role in the plant life cycle as a mechanism for releasing excess energy [34]. Additionally, most secondary metabolites play an essential role in increasing the resistance of plants to biotic and abiotic stresses. Therefore, the increase in the levels of phenolics, flavonoids, anthocyanins, and essential oil in field cultivation can be due to some limitations, such as unsuitable soil properties, unfavorable weather conditions, and lack of proper root access to water and nutrients compared to greenhouse cultivation [35]. In *Artemisia dracunculus* L., despite the decrease in vegetative growth, water deficit stress increased both the percentage and yield of essential oil, the contents of total phenolics, total flavonoids, and antioxidant properties compared to the control plants [5]. Jaafar et al. [36] reported that water deficit stress not only increased the total phenolic and flavonoid content of *Marantodes pumilum* (Blume) Kuntze but also increased their yield. In the comparison of parsley plants in different cultivation systems (field, greenhouse, and tunnel cultivation), the highest level of ascorbic acid was obtained in the field. The level of ascorbic acid in the field was 2.6 and 5.4 times higher than in tunnel cultivation and greenhouse cultivation, respectively [30]. A comparison of ginseng (*Panax ginseng* C.A.Mey.) seedlings cultivated in hydroponic and soil cultivation systems showed that total phenolic and flavonoid levels in soil culture were higher than in hydroponic culture [37]. The level of carlina oxide in *C. acaulis* plants grown in the field was significantly higher than in those grown under hydroponic conditions. The flavonoid content of plant leaves was similar between the two cultivation systems. However, the antioxidant capacity and concentration of secondary metabolites were higher in the soil cultivation system. The large difference in carlina oxide content between soil-grown and hydroponically grown plants was attributed to differences in plant growth conditions, such as water supply, nutritional status, temperature, relative humidity, and light intensity [15]. Braglia et al. [38] reported that total phenolic content and antioxidant activity were significantly higher in aquaponically grown basil plants compared to soil-grown organic basil plants.

In the present study, the highest levels of linalool, *δ*-cadinene, and spathulenol were observed in greenhouse soil cultivation. The level of 1,8-cineole was very low in the hydroponic culture. However, the level of methyl chavicol, the principal essential oil constituent, in hydroponic cultivation was higher than in field and greenhouse soil cultivations. Generally, most medicinal and industrial applications of basil depend on its essential oil, especially the methyl chavicol content. Methyl chavicol is commonly used as a flavoring agent in food and beverages [39]. Alvarenga et al. [40] showed that nutrition affects the essential oil content in yarrow plants in the hydroponic system. Low levels of Ca reduced the growth of *Achillea millefolium* L. more than those of other studied elements, while the removal of S, P, Mn, or B promoted the essential oil percentage. Moreover, their results showed that the removal of macronutrients or micronutrients may be beneficial, as it can boost the percentage of some volatile compounds. For example, the sabinene percentage increased with Mg removal and the cubebene percentage with P removal. The same can be observed by removing Zn, Fe, or Mo to promote the level of (*E*)-caryophyllene, a compound with anti-inflammatory activity. In the current study, the changes in the levels of basil essential compounds in different cultivation systems can be due to the change in the levels of nutrients available to the plant in different cultivation conditions.

Foliar application of micronutrients in each of the cultivation systems yielded different results. In the field, the highest plant height, stem dry weight, and leaf area were noted in Fe foliar application; in greenhouse soil cultivation, the highest plant height was observed with the application of B or Fe, the highest dry weight of the stem was observed with the application of Zn, and the largest leaf area was observed with the application of Mn; in hydroponic culture, the highest plant height was noted with the use of B. Overall, in field cultivation and hydroponic cultivation, the application of any micronutrient increased stem dry weight and leaf area compared to the control treatment. However, no significant change was observed among the elements. It seems that the difference in the level of nutrients in the field soil, greenhouse soil, and nutrient solution in the hydroponic system explains the different responses of basil to the foliar application of elements in different cultivation substrates. Micronutrients play a prominent role in the growth and metabolism of plants. Therefore, their deficiency may cause several physiological disorders in plants and thus reduce their yield and quality [41]. Considering the role of B in increasing the longitudinal growth and cell division in plants, plant growth and biomass production are expectedly enhanced following the application of B. In addition, Zn and B are particularly important in plant growth and development because they are essential in the synthesis of plant growth regulators [6]. The increase in plant growth parameters following the application of Fe, Mn, or Cu is also due to their vital role in many enzymes involved in physiological and biochemical processes [42].

In the present study, under field conditions, the highest levels of total phenolics, total flavonoids, and antioxidant capacity were observed following Zn or Mn foliar application. In hydroponic cultivation, foliar spraying of Zn or B led to the highest antioxidant capacity and levels of phenolics and flavonoids. Regardless of the cultivation system, foliar spraying of Cu, Mn, Zn, Fe, or B significantly increased basil anthocyanin content. Micronutrients such as Zn and Fe play an effective role in preserving antioxidants and increasing anthocyanin content. Additionally, Mn plays a very important role in the production of anthocyanins [43]. It has been reported that Fe, Mn, and Zn foliar application increased the total phenolic content of *Borago officinalis* L. [44]. Nutrient accessibility in the culture medium is an important factor in changing the yield and quality of the essential oil. Planting methods, climatic conditions, fertilization, irrigation, and harvesting date of plant material can change the yield and composition of the essential oil [45]. In the present study, regardless of the cultivation system, the highest essential oil percentage was noted in the treatment of foliar spraying with Zn or Mn. In field conditions and greenhouse soil cultivation, Zn, followed by Mn, foliar application led to the highest yield of essential oil. In hydroponic cultivation, the highest essential oil yield was observed following Mn, followed by Zn, foliar application. The highest level of methyl chavicol was found in hydroponic cultivation in all micronutrient treatments. In field conditions, Mn and B foliar spray resulted in the production of higher levels of methyl chavicol than other treatments. Considering the effects of B and Mn on plant growth and development, a reason for the increased levels of methyl chavicol in the essential oil seems to be the increased photosynthetic activity and the role of these elements in the chloroplast structure, which can lead to more production of essential oil secreting glands in the leaves [39]. Foliar spraying of Cu or Zn in the field also led to a significant increase in the level of cineol. In the greenhouse soil culture, the highest level of *epi-α*-cadinol was obtained in the essential oil of plants treated with Zn.

Kumara et al. [46] reported that treatment with Zn or Fe improved the quality of essential oil and increased the concentration of the main compounds of *Matricaria chamomilla* L. essential oil, such as chamazulene, *Z*-spiroether, and *α*-bisabolol oxide A and B. In agreement with the results of the present study, foliar spraying of Zn or B significantly enhanced the biomass, percentage and yield of essential oil, total phenolics, flavonoids, and antioxidant capacity of *S. khuzistanica*. However, the spraying of Zn had no effect on carvacrol, the dominant component of *S. khuzistanica* essential oil [47]. The root yield of ginseng, total ginsenoside content, and nine types of ginsenosides in roots were significantly increased under foliar application of Fe, Zn, Mn, or Cu. In this study, the concentration of 500 mg/L of Fe, Mn, Cu, or Zn led to the highest yield and quality of ginseng [48]. Zn contributes to the synthesis of tryptophan, a precursor of indole acetic acid, and access to appropriate levels of this micronutrient can increase crop yield and quality [6]. It also acts as a metal component of various enzymes or as a structural, functional, and regulatory cofactor in relation to carbohydrate metabolism, photosynthesis, and protein production [49]. Since glucose and CO_2_ are possible sources of carbon used in the biosynthesis of terpenes, the role of Zn in the production of essential oil seems to be very important. The spraying of micronutrients in mint plants caused a significant increase in plant growth and essential oil percentage and yield, as well as the level of menthol in the essential oil [50]. The foliar application of 50 mg/L of Mn or Zn and their combined application increased vegetative parameters, the essential oil percentage of seed, and the essential oil yield of the vegetative body and seed in cumin. The level of cumin-aldehyde, the dominant component of essential oil, also increased in seed essential oil and the vegetative body’s essential oil [51].

Foliar application of micronutrients did not have a significant effect on the level of macronutrients (N, Ca, P, Mg, and K). However, foliar spraying of each of the micronutrients increased their concentration in the aerial parts of basil. Nutrients applied via foliar spraying penetrate leaf tissue through the cuticle or stomata, leading to an increased concentration of certain micronutrients in the leaves. Foliar spraying of micronutrients is an effective method for addressing specific deficiencies that may not be fully resolved through soil or hydroponic solution applications alone [22,43]. Fe foliar application not only increased the concentration of Fe in aerial parts but also decreased the concentration of Cu compared to the control. Therefore, the increase in Fe concentration in plants had a negative effect on Cu absorption. Foliar spraying of different concentrations of chitosan in red amaranth had no effect on the absorption of nutrients Ca, Mg, Na, K, and P but caused a significant increase in the absorption of S, Zn, and Fe [52]. Furthermore, manganese sulfate spraying on pomegranate shrubs increased the concentration of Mn and N in the leaves, while foliar spraying of zinc sulfate increased the concentration of Zn and decreased those of Mn and P in the leaves [53]. In chickpea plants, soil application or foliar application of Fe decreased the absorption and concentration of Mn in the aerial parts of the plant [42].

## 4. Materials and Methods

### 4.1. Planting Plants, Growing Conditions, and Applying Treatments

The present research was carried out in the farm and in the research greenhouse of the College of Agriculture and Natural Resources of Lorestan University over two consecutive years, 2020 and 2021 (longitude 48°26′ E, latitude 33°44′ N, altitude 1170 m). In this study, basil plants were compared in three cultivation systems, including cultivation in the field, cultivation in soil pots, and drip hydroponic cultivation. In each of the cultivation systems, foliar spraying of micronutrients was applied in a randomized complete block design. There were six treatments (control [water spray], Fe, Mn, Cu, Zn, and B foliar application), each with three replications. Perlite was used as the substrate in hydroponic cultivation (pH = 7.2, EC = 1.5). The sources of microelements were iron sulfate, manganese sulfate, copper sulfate, zinc sulfate, and boric acid. Before planting the plants, soil samples from the pots and the field were collected and analyzed for physical and chemical characteristics, as shown in Table 6 [54].

Basil seeds (purchased from Pakan Seed Co., Isfahan, Iran) were cultivated in a tray at a temperature of 18–22 °C and a relative humidity of 60% with 12 h of light and 12 h of darkness. After 3 weeks (May 5), the four-leaf seedlings of basil plants were transferred to the 5 L pots of the hydroponic system (perlite), the 5 L pots for the soil bed greenhouse cultivation, and the field cultivation. The plants in the hydroponic system were fed with half-strength Hoagland’s solution with a pH = 6, adjusted with HCL or NaOH (0.5 mol L^−1^) (Table 7). For soil and hydroponic greenhouse cultivation, six pots were grown in each experimental unit. During the experiment, the average daily temperature of the greenhouse was 22–28 °C, the relative humidity was 60–70%, and the light intensity was 600 μmol/m^2^ per second. For the field cultivation, six plots of 2 × 2 m^2^ were prepared in each experimental block, each with 4 rows 50 cm apart. The plants were cultivated in the rows 20 cm apart. The distance between the plots was 1 m, and the distance between the blocks was 2 m. After planting, irrigation was carried out twice a week in the open field and soil culture in the greenhouse. Spraying of microelements at a concentration of 0.1% was conducted in all cultivation systems identically at 3 stages (6–8 real leaves, late vegetative growth, and the beginning of flowering) in the evening.

### 4.2. Reagents, Nutrients and Standards

Folin-Ciocalteu reagent, saturated sodium carbonate, aluminum chloride, potassium acetate, acetate buffer, ferric chloride, and iron sulfate were supplied by Sigma-Aldrich (St. Louis, MO, USA). Analytical grade 2,4,6-tripyridyl-s-triazine (TPTZ), 2,2-diphenyl-1-picrylhydrazyl (DPPH), hydrochloric acid, gallic acid, and rutin were purchased from Merck (Darmstadt, Germany).

Iron sulfate 99%, manganese sulfate 99.9%, copper sulfate 99.9%, zinc sulfate 99.9%, boric acid 99.9%, and Hoagland nutrient solution were obtained from Pars Oxide CO., Shiraz, Iran. 

### 4.3. Measurement of Plant Biomass

At the end of the experiment and in each cultivation system, five plants in each replication were randomly selected, and the plant height was measured. Four fully mature leaves were separated from each plant (16 leaves per treatment) to measure the leaf area, and their average was measured by a leaf area meter. Then, select samples were taken, and their fresh weight was recorded. The samples were placed in an oven at 70 °C for 48 h, and then their dry weight was recorded.

### 4.4. Measurement of Leaf Mineral Elements

The leaf samples were subjected to mineralization following the AOAC [55] procedure. Initially, the dried samples were placed in a furnace and heated at 500 °C for 2 h, resulting in the formation of ash. The ash was then dissolved in 4 mL of diluted HNO_3_ (1:1 ratio) with a density of 1.33. The solution was evaporated using a hot plate at 100 °C, leaving behind a residue. The residue was further heated at 500 °C for 1 h and allowed to cool. Subsequently, 10 mL of diluted HCl (1:1 ratio) with a density of 1.18 was added to the residue, and heat (100 °C) was applied on a hot plate to dissolve the residue. The concentration of phosphorus (P) in the resulting digest was measured using a spectrophotometer at a wavelength of 470 nm. Nitrogen (N) content was determined using the Kjeldahl method as described by AOAC [56]. Furthermore, the concentrations of potassium (K), calcium (Ca), magnesium (Mg), Mn, Fe, Zn, and Cu were measured using atomic absorption spectrometry (model 240FS, Agilent, Santa Clara, CA, USA), following the guidelines provided by AOAC [55]. Each treatment was replicated three times to ensure the accuracy and reliability of the results.

### 4.5. Essential Oil Isolation

A total of 15 g of dried basil aerial parts were hydrodistilled for 3 h with a Clevenger-type apparatus. The essential oil obtained (three replications) was weighed with a digital scale with an accuracy of 0.0001 g and dehydrated with dry sodium sulfate. The essential oil percentage in each sample was calculated according to Equation (1). The yield of essential oil was obtained by multiplying the percentage of essential oil with the dry weight of the aerial parts (gr/plant) [57].

Essential oil percentage% = (Essential oil weight/dry weight of plant) × 100(1)

### 4.6. GC/FID and GC/MS Analysis

The essential oils were subjected to analysis using gas chromatography (GC) in FID mode with a Thermo-UFM ultra-fast system (Agilent Scientific Instruments, USA). Separation was achieved using an HP-5 column (30 m length, 0.1 mm inner diameter, 0.40 μm film thickness). The carrier gas helium was utilized, and both the detector and injector (FID) were set to a temperature of 285 °C. The flow rate of the carrier gas was maintained at 1.1 mL/min, with a split ratio of 1:60. The oven temperature started at 60 °C for 5 min and then increased to 280 °C at a rate of 7 °C/min. The analysis was performed in triplicate.

For further characterization, a Varian 3400 GC/MS system was employed (Agilent Scientific Instruments, Santa Clara, CA, USA). A DB-5 fused silica column (30 m length, 0.25 mm inner diameter, 0.25 μm film thickness) was used. The carrier gas helium was maintained at a flow rate of 1 mL/min, and the split ratio was set to 1:50. The transfer line temperature was set to 260 °C. Flame ionization detection was performed with a linear velocity of 31.5 cm/s and a voltage of 70 eV, covering a mass range of 50–450 amu. The oven temperature was programmed to increase from 50 to 240 °C at a rate of 4 °C/min [6].

The identification of essential oil components was accomplished by comparing their retention indices (RI) and mass spectra with those in Wiley, Adams, and NIST05 libraries, as well as references in the literature [58]. Additionally, co-injection with available authentic standards was utilized. The relative percentages of the essential oil constituents were determined by normalizing the peak areas obtained from GC-FID analysis [59,60].

### 4.7. Anthocyanins

The Wagner [61] method was used to measure leaf anthocyanins. The leaf samples were thoroughly ground in a porcelain mortar containing an acidic methanol solution, and the obtained extract was placed in the dark for 24 h. After that, the samples were centrifuged, and the absorbance of the supernatant solution was recorded using a spectrophotometer at a wavelength of 550 nm; the content of anthocyanin was calculated in micromoles per g of fresh leaf weight.

### 4.8. Preparation of Methanol Extract and Measurement of Total Phenolics and Flavonoids

A total of 1 g of the dried basil leaves (harvested in the early stages of flowering) was placed in a magnetic shaker with 25 mL of 80% methanol for 3 h. The samples were then centrifuged at 3000 rpm for 5 min, and the supernatant solution was collected to measure its antioxidant properties [62].

The content of total phenolics in the extracts was measured based on the Folin–Ciocâlteu method [63]: 100 mL of the extract with a concentration of 1 mg/mL was added to 500 mL of Folin’s reagent. After 1 min, 1.5 mL of sodium bicarbonate 20% was added to each tube and then vortexed and incubated for 120 min at room temperature. The absorbance of the sample at 760 nm was read with a spectroscopic device. The standard curve was prepared by solutions of 50 to 500 mg/L of gallic acid in methanol. Total phenolic content was expressed as gallic acid equivalent (mg of gallic acid/g dry weight), which is a reference compound for determining phenolic content.

The flavonoid content was assessed using the aluminum chloride colorimetric method described by Chang et al. [64]. A total of 0.5 mL of each extract was combined with 1.5 mL of methanol. To this mixture, 0.1 mL of 10% aluminum chloride, 0.1 mL of 1 M potassium acetate, and 2.8 mL of distilled water were added. The resulting solution was incubated at room temperature for 30 min, and its absorbance at 415 nm was read by a spectrophotometer. Different concentrations of quercetin, 12.5–100 µg/mL in methanol, were used to draw a standard curve, and the dry extract content was expressed. Flavonoids were expressed as quercetin equivalent/g dry weight.

### 4.9. Evaluation of Antioxidant Properties

#### 4.9.1. Free Radical Inhibition (DPPH)

In this method, 3.9 mL of a prepared DPPH (2,2-diphenyl-1-picrylhydrazyl) stock solution was added to a test tube. The DPPH stock solution was prepared by dissolving 0.004 g of DPPH in 100 mL of methanol. Then, 0.1 mL of each extract was added to the test tube containing the DPPH solution. The test tube was placed in a dark environment for 30 min. The absorbance of the solution was read at a wavelength of 517 nm. DPPH radical inhibition percentage was calculated using Equation (2). Then the results were expressed as IC50 (an amount of antioxidant that is necessary for the concentration of DPPH to reach 50% of the initial value) [65].

I% = [(A_0_ − As)/A_0_] * 100 (2)

where I% represents free radical inhibition percentage, A_0_ represents control absorption, and As represents sample absorption.

#### 4.9.2. Ferric Reducing Antioxidant Potential (FRAP)

Antioxidants that have the ability to reduce Fe^3+^ to Fe^2+^ and change the colorless TPTZ-Fe^3+^ complex to the blue TPTZ-Fe^2+^ complex, whose intensity can be measured at the wavelength of 593 nm. For this purpose, the concentration of 250 µg/mL of plant extract was taken and added to the final volume of 2 mL of FRAP solution containing TPTZ 10 mM (in 40 mM HCl), 20 mM iron chloride, and 300 mM acetate buffer at pH = 3.6. The above sample was placed at a temperature of 37 °C for 10 min, and the intensity of the resulting color was read at a wavelength of 593 nm against a blank. To draw the standard curve for the FRAP method, iron sulfate (FeSO_4_, 7H_2_O) with concentrations of 1000, 500, 250, and 125 μM was used, and the antioxidant capacity of the extracts was expressed as micromoles of Fe^2+^ per gram of fresh weight of plant [66].

### 4.10. Statistical Calculations

The mean of data obtained from the 2-year research was subjected to variance analysis according to the compound analysis, and the comparison of means was conducted with the LSD test at *p* ≤ 0.05. Excel was used in order to draw graphs. 

## 5. Conclusions

In the current study, basil plants grown in a hydroponic system had higher yields, which was due to the continuous supply of nutrients such as K, Ca, Mn, N, P, Mn, Fe, B, and Zn in this system. Field-grown basil plants had more woody growth and showed a higher stem dry weight. They also had higher levels of essential oil, phenolics, flavonoids, anthocyanins, and antioxidant activity. Therefore, in terms of biological properties and the content of bioactive components, field cultivation of basil is more suitable than hydroponic cultivation. However, there was no significant difference in the yield of essential oil in field cultivation and hydroponic systems. The level of methyl chavicol, the dominant component of basil essential oil, was higher in hydroponic culture, while the percentage of 1,8-cineole in the essential oil was very low in this system. The highest levels of linalool, *epi-α*-cadinol, *δ*-cadinene, and spathulenol were found in the greenhouse soil culture. It can be stated that the essential oils of plants grown in the hydroponic system had a higher economic value due to the higher levels of methyl chavicol. 

Foliar spraying of Cu, Mn, Zn, Fe, and B significantly increased the leaf dry weight and anthocyanin content of basil. However, foliar application of micronutrients in each of the cultivation systems led to different results. In field conditions, the highest levels of total phenolics, total flavonoids, and antioxidant capacity were observed following Zn or Mn foliar application. In hydroponic cultivation, foliar spraying of Zn or B led to the highest levels of antioxidant activity, phenolics, and flavonoids. In all three cultivation systems, foliar application of B, Mn, or Zn led to the highest percentage and yield of essential oil. It seems that the difference in the level of nutrients in the field soil, greenhouse soil, and nutrient solution in the hydroponic system is the reason for the different responses of plants to the foliar application of elements in different cultivation substrates.

## Figures and Tables

**Table 1:** Mean comparison of the interaction effect of cultivation system and foliar spraying on biomass and phytochemical characteristics of basil.

Cultivation System	Foliar Spray	Plant Height (cm)	Leaf Area (cm^2^)	Stem Dry Weight (g)	Essential Oil Yield (g/plant)	Total Phenol (mg GAE/g DW)	Total Flavonoids (mg Q/g DW)	DPPH (IC_50_ mg/mL)	FRAP (mmol Fe/g DW)
Field	Control	31.07 ± 1.40 ^j^	5.87 ± 0.62 ^h^	8.86 ± 0.72 ^cd^	0.03 ± 0.002 ^ij^	6.42 ± 1.02 ^de^	3.61 ± 1.02 ^efg^	7.57 ± 1.03 ^c^	3.49 ± 0.42 ^ef^
	Cu	37.84 ± 1.20 ^i^	9.34 ± 0.43 ^g^	14.54 ± 1.46 ^a^	0.06 ± 0.003 ^g–j^	8.31 ± 1.33 ^cd^	4.79 ± 1.21 ^de^	6.46 ± 1.12 ^efd^	5.60 ± 0.67 ^b^
	Zn	37.44 ± 2.60 ^i^	9.39 ± 0.72 ^g^	13.62 ± 1.83 ^a^	0.30 ± 0.05 ^a^	16.54 ± 2.42 ^a^	9.56 ± 2.01 ^a^	5.41 ± 1.1 ^g^	7.26 ± 1.02 ^a^
	Mn	40.08 ± 3.46 ^hi^	8.93 ± 1.33 ^g^	13.86 ± 2.13 ^a^	0.16 ± 0.04 ^cde^	12.06 ± 1.91 ^b^	7.17 ± 1.44 ^b^	5.84 ± 0.87 ^fg^	7.15 ± 1.14 ^a^
	Fe	41.87 ± 2.91 ^gh^	9.56 ± 1.33 ^g^	14.56 ± 2.22 ^a^	0.14 ± 0.04 ^d–g^	12.11 ± 2.03 ^b^	6.58 ± 1.84 ^bc^	6.63 ± 1.23 ^efd^	5.41 ± 1.1 ^b^
	B	39.50 ± 4.32 ^hi^	8.90 ± 0.82 ^g^	14.37 ± 2.33 ^a^	0.18 ± 0.02 ^cd^	7.58 ± 1.12 ^cde^	4.01 ± 1.15 ^d–g^	5.99 ± 1.42 ^efg^	7.08 ± 1.13 ^a^
Greenhouse soil culture	Control	45 ± 4.24 ^fg^	11.31 ± 1.43 ^f^	4.25 ± 0.54 ^e^	0.02 ± 0.004 ^j^	5.99 ± 1.01 ^e^	2.45 ± 0.72 ^fgh^	8.56 ± 1.68 ^b^	3.94 ± 0.54 ^de^
	Cu	44.46 ± 3.33 ^fg^	12.08 ± 12 ^def^	12.1 ± 0.95 ^ab^	0.03 ± 0.006 ^ji^	8.34 ± 1.35 ^cd^	5.46 ± 1.44 ^cd^	6.42 ± 1.51 ^def^	2.96 ± 0.84 ^fg^
	Zn	44.5 ± 3.65 ^fg^	13.31 ± 1.31 ^c^	14.06 ± 1.54 ^a^	0.13 ± 0.03 ^d–g^	5.62 ± 0.82 ^e^	3.80 ± 0.73 ^e–h^	6.87 ± 1.43 ^cde^	2.95 ± 0.63 ^fg^
	Mn	47 ± 3.54 ^ef^	13.84 ± 1.41 ^bc^	7.87 ± 0.74 ^cd^	0.10 ± 0.02 ^e–j^	7.15 ± 1.55 ^cde^	4.02 ± 1 ^e–h^	6.76 ± 063 ^cde^	5.36 ± 0.86 ^b^
	Fe	50.33 ± 4.71 ^d^	12.85 ± 1 ^cde^	12.05 ± 1.54 ^ab^	0.06 ± 0.008 ^hi^	8.98 ± 1.43 ^c^	4.45 ± 1.32 ^de^	6.99 ± 1.02 ^cd^	3.50 ± 0.62 ^ef^
	B	54.66 ± 4.36 ^c^	11.89 ± 1.31 ^ef^	9.78 ± 0.91 ^cd^	0.1 ± 0.04 ^e–i^	7.02 ± 0.91 ^cde^	2.32 ± 0.67 ^ghi^	6.86 ± 1.1 ^cde^	4.97 ± 0.89 ^bc^
Hydroponic culture	Control	49.38 ± 4.22 ^cd^	13.1 ± 1.61 ^cd^	6.48 ± 0.72 ^de^	0.03 ± 0.01 ^hij^	1.57 ± 0.42 ^f^	0.64 ± 0.12 ^i^	10.48 ± 2.01 ^a^	2.17 ± 0.07 ^g^
	Cu	60.45 ± 5.32 ^ab^	14.53 ± 2.31 ^ab^	8.76 ± 0.82 ^cd^	0.07 ± 0.02 ^f–j^	2.40 ± 0.62 ^f^	0.87 ± 0.05 ^hi^	10.25 ± 1.7 ^a^	3.02 ± 0.64 ^fg^
	Zn	58.05 ± 5.33 ^ab^	14.72 ± 2.32 ^ab^	9.81 ± 1.46 ^bc^	0.22 ± 0.03 ^cb^	6.78 ± 1.47 ^cde^	4.19 ± 1.01 ^def^	6.95 ± 1.2 ^cd^	4.36 ± 0.9 ^cde^
	Mn	58.81 ± 3.24 ^ab^	15.33 ± 2.42 ^a^	8.53 ± 1.16 ^cd^	0.27 ± 0.05 ^ab^	2.64 ± 0.18 ^f^	1.26 ± 0.22 ^hi^	6.63 ± 1.02 ^def^	2.95 ± 0.8 ^fg^
	Fe	57.63 ± 3.91 ^b^	14.51 ± 1.53 ^ab^	9.37 ± 1.41 ^bcd^	0.11 ± 0.02 ^e–h^	5.63 ± 1.02 ^e^	4.13 ± 0.55 ^def^	6.55 ± 0.78 ^def^	2.87 ± 0.64 ^fg^
	B	60.93 ± 3.56 ^a^	15.13 ± 2.4 ^2a^	8.47 ± 0.82 ^cd^	0.15 ± 0.03 ^e–d^	7.60 ± 1.66 ^cde^	4.17 ± 1.01 ^def^	8.52 ± 1.32 ^b^	4.41 ± 0.88 ^cd^

Means with the same letters in each column do not have a significant difference at the 1% probability level based on the LSD test.

**Table 2:** Mean comparison of the effect of the cultivation system on the amount of some macroelements and microelements in basil leaves.

Cultivation System	N (%)	K (%)	P (%)	Mg (%)	Ca (%)	Cu (µg/g)	Zn (µg/g)	Fe (µg/g)	Mn (µg/g)	B (µg/g)
Field	3.36 ± 0.44 ^b^	0.60 ± 0.15 ^c^	0.36 ± 0.03 ^b^	0.22 ± 0.04 ^b^	0.35 ± 0.05 ^b^	6.50 ± 0.82 ^b^	27.26 ± 1.76 ^c^	58.16 ± 4.47 ^c^	29.74 ± 3.88 ^c^	4.36 ± 1.12 ^b^
Greenhouse soil culture	3.41 ± 0.38 ^b^	0.62 ± 0.14 ^b^	0.52 ± 0.05 ^b^	0.23 ± 0.05 ^b^	0.37 ± 0.06 ^b^	7.94 ± 1.48 ^a^	41.95 ± 2.45 ^b^	64.76 ± 6.95 ^b^	44.18 ± 6.14 ^b^	4.53 ± 0.88 ^b^
Hydroponic culture	4.06 ± 0.67 ^a^	1.25 ± 0.28 ^a^	0.60 ± 0.17 ^a^	0.29 ± 0.03 ^a^	0.58 ± 0.06 ^a^	6.50 ± 1.32 ^b^	53.36 ± 3.94 ^a^	74.08 ± 8.75 ^a^	94.3 ± 8.63 ^a^	5.66 ± 1.44 ^a^

Means with the same letters in each column do not have a significant difference at the 1% probability level based on the LSD test.

**Table 3:** Mean comparison of the effect of foliar spraying of micronutrients on the concentration of some microelements in basil leaves.

Foliar Spray	Cu	Zn	Fe	Mn	B
(µg/g)					
Control	7.03 ± 2.11 ^b^	38.12 ± 3.55 ^bc^	61.43 ± 6.44 ^b^	51.64 ± 3.74 ^b^	4.88 ± 1.38 ^b^
Cu	12.91 ± 2.38 ^a^	33.38 ± 4.21 ^c^	59.56 ± 6.65 ^b^	49.05 ± 6.43 ^b^	4.28 ± 1.22 ^b^
Zn	7.23 ± 1.34 ^b^	54.02 ± 4.75 ^a^	60.34 ± 7.74 ^b^	48.83 ± 4.65 ^b^	4.30 ± 1.11 ^b^
Mn	6.59 ± 1.14 ^b^	42.18 ± 5.78 ^b^	58.42 ± 4.73 ^b^	90.30 ± 8.62 ^a^	4.29 ± 0.84 ^b^
Fe	6.35 ± 1.46 ^b^	36.52 ± 4.66 ^bc^	94.95 ± 8.37 ^a^	46.98 ± 7.55 ^b^	4.30 ± 0.78 ^b^
B	7.30 ± 1.55 ^b^	40.89 ± 4.26 ^bc^	59.33 ± 6.36 ^b^	50.67 ± 7.71 ^b^	7.05 ± 2.08 ^a^

Means with the same letters in each column do not have a significant difference at the 1% probability level based on the LSD test.

**Table 4:** Constituents of the essential oil in basil.

No	Oil Constituents	RI ^a^	LIT RI ^b^	ID ^c^	No	Oil Constituents	RI	KI	ID ^c^
1	*β*-Myrcene	988	987.03	RI, MS	16	*γ*-Muurolene	1449	1454.83	RI, MS
2	1,8-Cineole	1031	1030.11	Std	17	*Z*-*β*-Farnesene	1460	1458.14	RI, MS
3	(*E*)-*β*-Ocimene	1040	1041.43	RI, MS	18	Germacrene D	1474	1473.74	Std
4	Fenchone	1088	1094.28	RI, MS	19	*α*-Selinene	1498	1500	RI, MS
5	Linalool	1101	1102.99	Std	20	*α*-Bulnesene	1509	1495.36	RI, MS
6	Camphor	1146	1150.07	RI, MS	21	*γ*-Cadinene	1514	1506.84	RI, MS
7	Borneol	1164	1166.13	RI, MS	22	*δ*-Cadinene	1525	1512.63	RI, MS
8	Terpinen-4-ol	1177	1184.16	RI, MS	23	(*E*)-Nerolidol	1554	1557.69	RI, MS
9	Methyl chavicol	1200	1207.62	Std	24	Spathulenol	1572	1568.52	RI, MS
10	*β*-Copaene	1378	1370.31	RI, MS	25	Caryophyllene oxide	1573	1571.84	Std
11	Bornyl acetate	1283	1283.18	RI, MS	26	Isoaromadendrene epoxide	1579	1574.05	RI, MS
12	Methyl eugenol	1400	1402.32	Std	27	1,10-di-*epi*-Cubenol	1601	1606.67	RI, MS
13	(*E*)-Caryophyllene	1415	1413.84	RI, MS	28	*epi*-*α*-Cadinol	1644	1635.45	RI, MS
14	*α*-Bergamotene	1430	1427.91	RI, MS	29	*β*-Eudesmol	1649	1644.82	RI, MS
15	*β*-Gurjunene	1449	1443.71	RI, MS	30	*α*-Bisabolol	1691	1681.46	RI, MS

^a^ Linear retention indices on the HP-5 column, which were experimentally determined using a homologous series of n-alkanes. ^b^ Relative retention indices, which were taken from Adams. ^c^ Identification methods: MS, by comparison of the mass spectrum with those of the computer mass libraries (Wiley, Adams, and NIST 05); RI: by comparison of the retention index with those reported in the literature; Std: by comparison of the retention time and mass spectrum of an available authentic standard.

**Table 5:** Mean comparison of the interaction effect of cultivation system and foliar spraying on basil essential oil constituents.

Cultivation System	Foliar Spray	1,8-Cineole	Methyl Chavicol	*α*-Bergamotene	*γ*-Cadinene	Caryophyllene Oxide	1,10-di-*epi*-Cubenol	*epi*-*α*-Cadinol
Field	Control	1.41 ± 0.38 ^de^	43.08 ± 2.72 ^bcd^	2.62 ± 0.58 ^a–d^	0.95 ± 0.08 ^b^	1.34 ± 0.03 ^b^	1.14 ± 0.06 ^bc^	8.89 ± 1.03 ^def^
	Cu	6.29 ± 1.08 ^a^	40.09 ± 4.62 ^cde^	2.61 ± 0.67 ^a–d^	0.8 ± 0.06 ^b^	0.69 ± 0.02 ^bc^	0.96 ± 0.04 ^bc^	6.57 ± 1.02 ^fgh^
	Zn	6.80 ± 1.11 ^a^	41.49 ± 3.77 ^b–e^	2.69 ± 0.85 ^a–d^	0.93 ± 0.05 ^b^	1.16 ± 0.28 ^bc^	1.27 ± 0.13 ^bc^	7.86 ± 1.11 ^efg^
	Mn	4.50 ± 0.58 ^b^	51.62 ± 5.62 ^abc^	2.63 ± 0.93 ^a–d^	0.52 ± 0.03 ^b^	0.77 ± 0.13 ^bc^	0.72 ± 0.15 ^c^	5.03 ± 0.58 ^hi^
	Fe	2.79 ± 0.28 ^cd^	24.21 ± 3.55 ^f^	3.04 ± 1.01 ^ab^	2.03 ± 0.28 ^a^	2.26 ± 0.14 ^a^	5.10 ± 1.01 ^a^	10.37 ± 1.44 ^cde^
	B	3.66 ± 0.73 ^bc^	46.32 ± 5.72 ^abc^	1.62 ± 0.28 ^c–f^	0.69 ± 0.04 ^b^	1.06 ± 0.08 ^bc^	1.11 ± 0.04 ^bc^	5.89 ± 1.02 ^ghi^
Greenhouse soil culture	Control	0.87 ± 0.08 ^e^	30.21 ± 4.11 ^ef^	1.92 ± 0.25 ^b–f^	2.65 ± 0.38 ^a^	1.31 ± 0.09 ^b^	2.57 ± 0.12 ^b^	9.42 ± 1.04 ^de^
	Cu	0.66 ± 0.12 ^e^	31.09 ± 4.45 ^def^	2.67 ± 0.28 ^a–d^	0.41 ± 0.08 ^b^	1.12 ± 0.05 ^bc^	1.32 ± 0.04 ^bc^	18.88 ± 2.52 ^b^
	Zn	0.60 ± 0.18 ^e^	27.15 ± 3.32 ^f^	1.36 ± 0.22 ^ef^	0.34 ± 0.03 ^b^	0.55 ± 0.03 ^c^	1.73 ± 0.16 ^bc^	24.01 ± 3.78 ^a^
	Mn	0.81 ± 0.09 ^e^	32.34 ± 3.31 ^def^	1.59 ± 0.29 ^def^	0.78 ± 0.09 ^b^	1.32 ± 0.18 ^b^	1.74 ± 0.48 ^bc^	12.17 ± 1.88 ^c^
	Fe	0.61 ± 0.05 ^e^	31.83 ± 2.61 ^def^	1.29 ± 0.34 ^ef^	0.67 ± 0.12 ^b^	1.06 ± 0.11 ^bc^	1.8 ± 0.23 ^bc^	11.15 ± 1.95 ^cd^
	B	0.61 ± 0.11 ^e^	33.2 ± 4.63 ^def^	1.40 ± 0.38 ^ef^	2.2 ± 0.23 ^a^	0.69 ± 0.05 ^bc^	1.13 ± 0.14 ^bc^	17.08 ± 2.58 ^b^
Hydroponic culture	Control	0.62 ± 0.06 ^e^	51.06 ± 5.64 ^abc^	3.76 ± 1.01 ^a^	0.86 ± 0.05 ^b^	1.05 ± 0.07 ^bc^	0.93 ± 0.11 ^bc^	5.58 ± 0.56 ^ghi^
	Cu	0.6 ± 0.12 ^e^	55.86 ± 5.75 ^ab^	2.45 ± 0.08 ^b–e^	1.03 ± 0.08 ^b^	0.84 ± 0.03 ^bc^	0.58 ± 0.08 ^c^	3.44 ± 0.78 ^i^
	Zn	0.66 ± 0.07 ^e^	57.36 ± 5.96 ^a^	2.82 ± 0.09 ^abc^	0.6 ± 0.04 ^b^	0.98 ± 0.08 ^bc^	0.72 ± 0.09 ^c^	4.75 ± 1.04 ^hi^
	Mn	0.6 ± 0.11 ^e^	52.14 ± 6.75 ^abc^	2 ± 0.25 ^b–f^	0.87 ± 0.06 ^b^	0.96 ± 0.06 ^bc^	0.77 ± 0.12 ^bc^	5.51 ± 0.48 ^ghi^
	Fe	0.67 ± 0.13 ^e^	58.54 ± 6.84 ^a^	1.9 ± 0.18 ^b–f^	0.4 ± 0.02 ^b^	1.08 ± 0.13 ^bc^	0.43 ± 0.04 ^c^	3.78 ± 0.45 ^i^
	B	0.67 ± 0.10 ^e^	53.64 ± 4.81 ^ab^	2.34 ± 0.32 ^b–e^	0.77 ± 0.18 ^b^	0.69 ± 0.04 ^bc^	0.88 ± 0.09 ^bc^	3.67 ± 0.66 ^ghi^

Means with the same letters in each column do not have a significant difference at the 1% probability level based on the LSD test.

**Table 6:** Some physicochemical properties of field soil and pot substrate.

Parameters (Units)	Field Soil	Pot Substrate
pH	7.13	6.9
EC (dS m^−1^)	2.51	2.02
Organic matter content (%)	1.74	2.8
Organic carbon content (%)	1.04	1.4
Total nitrogen (%)	0.15	0.24
Potassium (mg kg^−1^)	331	362
Phosphorus (mg kg^−1^)	12.4	14.76
Sodium (mg kg^−1^)	78.2	66.8
Magnesium (mg kg^−1^)	538.8	603.6
Ferrite (mg kg^−1^)	5.18	4.44
Textural class	Sandy clay loam	Sandy clay loam

**Table 7:** Hoagland nutrient solution.

Compounds	MgSO_4_·7H_2_O	Ca(NO_3_)_2_·4H_2_O	KH_2_PO_4_	KNO_3_	H_3_BO_3_	MnCl_2_·4H_2_O	ZnSO_4_·7H_2_O	CuSO_4_·5H_2_O	NaMoO_3_	Fe-DTPA
Unit	mmol L^−1^									mg L^−1^
Concentration	2	5	1	5	1	1	1	1	1	50

## Data Availability

The datasets used and/or analyzed in this study are available from the corresponding author upon reasonable request.

## Supplementary Materials

The following supporting information can be downloaded at: https://www.mdpi.com/article/10.3390/plants13172498/s1, Table S1: Variance analysis of the effect of foliar spraying of some micronutrient and cultivation system on biomass and phytochemical characteristics of basil; Table S2: Variance analysis of the effect of cultivation system and foliar spraying of some micronutrient on the amount of micro and macro elements in basil leaves; Table S3: Variance analysis of the effect of cultivation system and foliar spraying of some micronutrient on essential oil constituents of basil.
