# Supplementary material for: Effects of Micronutrients on the Growth and Phytochemical Composition of Basil (Ocimum basilicum L.) in the Field and Greenhouse (Hydroponics and Soil Culture)
Source: Plants (Basel). 2024 Sep 6;13(17):2498. doi: 10.3390/plants13172498 (PMC11397607; doi:10.3390/plants13172498)
Supplement: Supplementary file 1 [file plants-13-02498-s001.zip › plants-3171807-supplementary.pdf]

Supplementary Table S1. Variance analysis of the effect of foliar spraying of some micronutrient and cultivation system on biomass and phytochemical characteristics of basil

S.O.V	Plant height	leaf area	Stem dry weight	Leaf dry weight	Essential oil percentage	Essential oil yield	Anthocyanin	Total phenol	Total flavonoids	DDPH	FRAP
Cultivation system (A)	1723.68**	161.29**	105.87**	96.63**	1.80**	0.031**	2.83**	166.16**	53.84**	16.68**	35.80**
Error a	3.57	0.59	3.84	2.84	0.08	0.001	0.51	1.19	2.91	0.15	0.26
Foliar spray (B)	31.67**	8.37**	42.99**	42.15**	3.46**	0.047**	1.36*	17.24**	8.92**	3.57**	4.90**
A*B	9.87**	1.00*	6.63*	5.23ns	0.19ns	0.005**	0.43ns	25.11**	10.14**	4.79**	3.58**
Error b	1.34	0.45	2.94	2.71	0.09	0.001	0.45	1.24	0.81	0.23	0.25

**: Significant at 1% level. *: Significant at 5%, ns: not statistically significant.

Supplementary Table S2. Variance analysis of the effect of cultivation system and foliar spraying of some micronutrient on the amount of micro and macro elements in basil leaves.

S.O.V	K	Ca	Mg	N	P	Cu	Zn	Fe	Mn	B
Cultivation system (A)	2.47**	0.27**	0.025**	2.74*	0.283*	34.26**	3082.08**	1151.97**	20663**	8.96*
Error a	0.700	0.006	0.000	0.302	0.002	0.4	18.98	56.46	28.34	0.06
Foliar spray (B)	0.012ns	0.005ns	0.001ns	0.146ns	0.002ns	55.32**	463.06**	1860.72**	2407.9**	11.0**
A*B	0.002ns	0.006ns	0.000ns	0.155ns	0.003ns	1.82ns	60.07ns	50.79ns	142.74ns	0.378ns
Error b	0.012	0.011	0.004	0.191	0.002	1.51	31.61	57.80	67.99	0.607

**: Significant at 1% level, *: Significant at 5% level, ns: not statistically significant.

Supplementary Table S3. Variance analysis of the effect of cultivation system and foliar spraying of some micronutrient on essential oil constituents of basil

S.O.V	1,8-Cineole	Linalool	Camphor	Methyl chavicol	α-Bergamotene	Germacrene-D	γ -Cadinene	δ-Cadinene	Spathulenol	Caryophyllene oxide	1,10-di-*epi*-Cubenol	*epi*-α-Cadinol
cultivation system (A)	7.354**	763.1**	59.54^{ns}	2567.7**	3.19**	0.35^{ns}	0.80**	1.24*	3.58**	0.33^{ns}	5.9**	591.41**
Error a	0.35	25.02	20.96	25.1	0.78	0.12	0.18	0.15	0.06	0.12	0.73	2.37
foliar spray (B)	4.66**	39.33^{ns}	52.38^{ns}	54.49^{ns}	1.04*	0.57^{ns}	1**	0.82^{ns}	0.06^{ns}	0.14*	2.50*	36.48**
A*B	5**	25^{ns}	48.13^{ns}	102.17*	1.4**	0.51^{ns}	1.32**	0.52^{ns}	0.15^{ns}	0.33*	2.5*	33.17**
Error b	0.93	16.41	22.16	44.82	0.4	0.35	0.13	0.30	0.10	0.12	0.85	1.82

**: Significant at 1% level. *: Significant at 5% level, ns: not statistically significant.
